# The ALS-inducing factors, TDP43^A315T^ and SOD1^G93A^, directly affect and sensitize sensory neurons to stress

**DOI:** 10.1038/s41598-018-34510-8

**Published:** 2018-11-08

**Authors:** Sydney K. Vaughan, Natalia M. Sutherland, Sihui Zhang, Theo Hatzipetros, Fernando Vieira, Gregorio Valdez

**Affiliations:** 1 Virginia Tech Carilion Research Institute, Roanoke, Virginia USA; 2 Graduate Program in Translational Biology, Medicine and Health, Virginia Tech, Blacksburg, Virginia USA; 3 Department of Biological Sciences, Virginia Tech, Blacksburg, Virginia USA; 4 ALS Therapy Development Institute, Cambridge, MA USA

## Abstract

There is increased recognition that sensory neurons located in dorsal root ganglia (DRG) are affected in amyotrophic lateral sclerosis (ALS). However, it remains unknown whether ALS-inducing factors, other than mutant superoxide dismutase 1 (SOD1^G93A^), directly affect sensory neurons. Here, we examined the effect of mutant TAR DNA-binding protein 1 (TDP43^A315T^) on sensory neurons in culture and *in vivo*. In parallel, we reevaluated sensory neurons expressing SOD1^G93A^. We found that cultured sensory neurons harboring either TDP43^A315T^ or SOD1^G93A^ grow neurites at a slower rate and elaborate fewer neuritic branches compared to control neurons. The presence of either ALS-causing mutant gene also sensitizes sensory neurons to vincristine, a microtubule inhibitor that causes axonal degeneration. Interestingly, these experiments revealed that cultured sensory neurons harboring TDP43^A315T^ elaborate shorter and less complex neurites, and are more sensitive to vincristine compared to controls and to SOD1^G93A^ expressing sensory neurons. Additionally, levels of two molecules involved in stress responses, ATF3 and PERK are significantly different between sensory neurons harboring TDP43^A315T^ to those with SOD1^G93A^ *in vitro* and *in vivo*. These findings demonstrate that sensory neurons are directly affected by two ALS-inducing factors, suggesting important roles for this neuronal subpopulation in ALS-related pathogenesis.

## Introduction

Amyotrophic Lateral Sclerosis (ALS) is an adult-onset neurodegenerative disease that causes paralysis and death within 5 years of diagnosis^1^. Although the disease has been clinically recognized for over 140 years, effective and long-term therapies for ALS have yet to be developed. However, research over the last few decades has provided important insights on ALS. It is now known that the etiology of ALS is diverse, with heritable mutations in several genes accounting for 10% of the disease and the other 90% due to non-familial causes^2^. These efforts have also revealed that ALS is a multi-system disorder even though the disease is invariably characterized by progressive degeneration of α-motor neurons and their axons in skeletal muscles^3^. In addition to α-motor neurons, ALS-inducing factors affect other cells, including glial cells^4–6^, Renshaw cells^7,8^, corticospinal neurons^9^ and parvalbumin-positive interneurons^10^. More recently, evidence has accrued indicating that sensory neurons located in dorsal root ganglia (DRG), and thus the peripheral nervous system, are also affected in ALS^11–18^. Sensory neurons send critical information from peripheral tissues to the central nervous system to modulate motor function^19^. In particular, proprioceptive sensory neurons constantly relay information regarding the functional status of skeletal muscles directly to α-motor neurons. Thus, the dysfunction or loss of these neurons would inevitably impair motor function, and potentially increase α-motor neurons’ susceptibility to ALS-inducing factors as shown to occur in spinal muscular atrophy^20,21^.

In individuals with sporadic and familial ALS, including those with mutations in the superoxide dismutase 1 (SOD1) gene, sensory neurons have been found to malfunction and degenerate^15,16,22^. Corroborating these findings, mutant SOD1 (SOD1^G93A^) was shown to accumulate in the soma of sensory neurons and to cause degeneration of their axons in mice^13,14,23^. Interestingly, the degeneration of sensory axons follows a “dying back” process that begins prior to the onset of ALS-related motor deficits^23^, largely mirroring the cellular changes found in α-motor neurons affected with ALS^24^. Despite this increased recognition that sensory neurons are affected in ALS, only SOD1^G93A^ has been shown to directly affect sensory neurons. Thus, it remains unknown if other ALS-causing mutant genes also cause intrinsic and long-lasting deleterious changes in sensory neurons. In addition to SOD1, mutations in the 43-kDa TAR DNA binding protein (TDP43), the RNA-binding protein FUS/TLS (FUS), the gene for chromosome 9 open reading frame 72 (C9ORF72), and several other genes are known to cause ALS^2^.

In this study, we asked if mutant TDP43^A315T^ directly affects sensory neurons. We also reexamined the effect of SOD1^G93A^ on sensory neurons. We found that cultured sensory neurons harboring either ALS-causing mutant gene regenerate neurites at a slower rate and are more sensitive to vincristine, a cellular stressor, compared to control neurons. The presence of TDP43^A315T^ or SOD1^G93A^ also reduced the complexity of neuritic arbors. Along with these cellular changes, sensory neurons harboring either ALS-causing mutant gene exhibit altered expression of ATF3 and PERK, two stress response genes, in culture and *in vivo*. These findings demonstrate that TDP43^A315T^, in addition to SOD1^G93A^, directly affect sensory neurons in culture and *in vivo*.

## Results

### TDP43^A315T^ and SOD1^G93A^ impair sensory neurons’ ability to extend neurites

**Figure 1:**
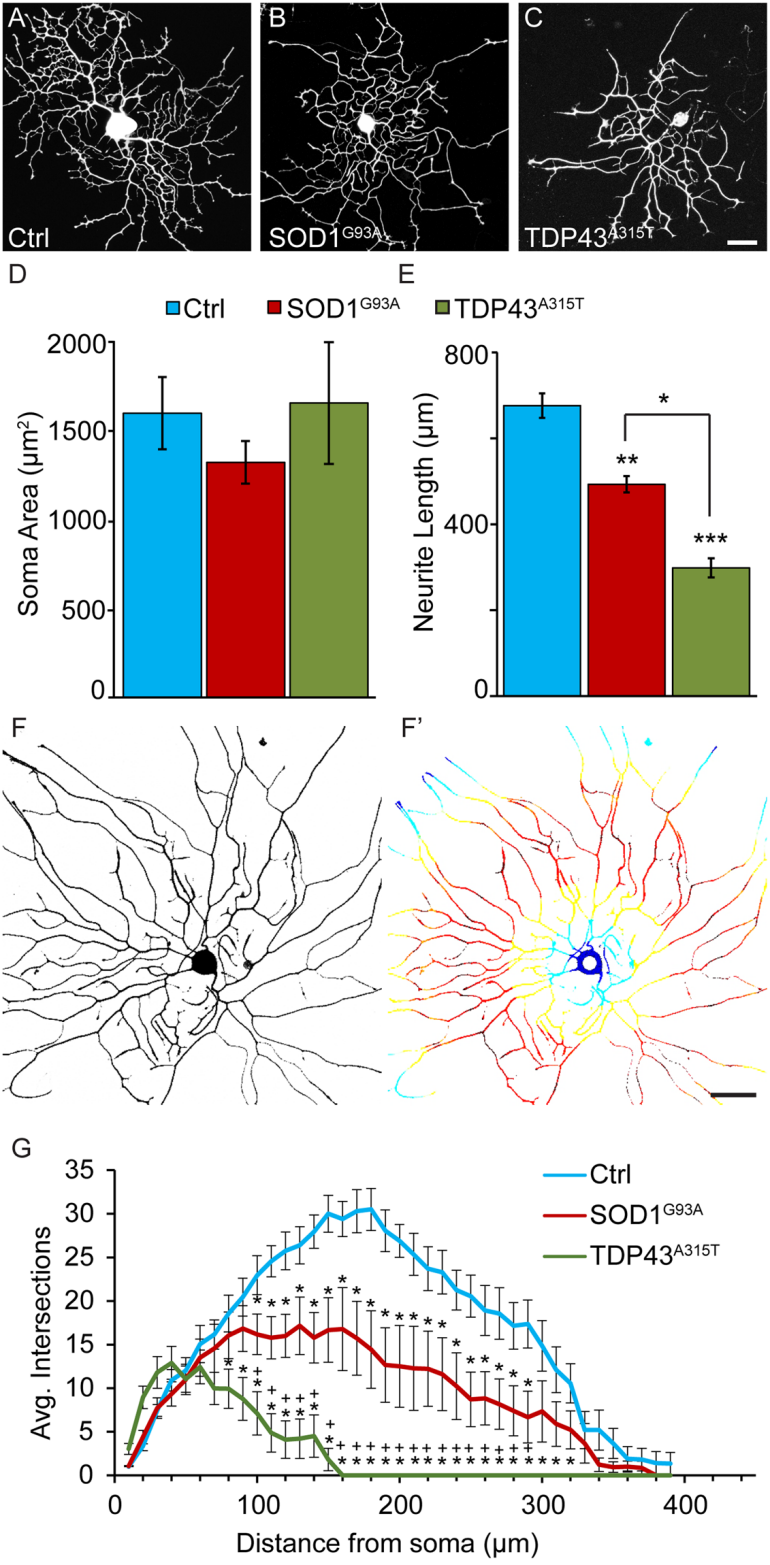
Decreased neurite length and complexity in SOD1^G39A^ and TDP43^A315T^ sensory neurons after 24 hours in culture. Cultures of dissociated sensory neurons were examined 24 hours post-plating. Control (**A**) and SOD1^G93A^ (**B**) mice were crossed with Thy1-YFP16 transgenic animals expressing YFP to visualize sensory neurons. Sensory neurons from TDP43^A315T^ animals were visualized using anti-β-tubulin (**C**). The soma area of sensory neurons remains consistent across genotypes (**D**). However, sensory neurons from SOD1^G93A^ and TDP43^A315T^ animals show significantly decreased neurite lengths (**E**) compared to control. Sholl analysis was performed using ImageJ where continuous concentric circles are drawn from the center of the neuron and expanding through the neuritic arbor (**F**–**F’**; colored rings). This analysis revealed that sensory neurons from SOD1^G93A^ and TDP43^A315T^ mice have significantly fewer neurite branches (**G**). Control n = 3; 130 days old, SOD1^G93A^ n = 3; 130 days old, and TDP43^A315T^ n = 4; 130 days old. Only male mice were used for this experiment. At least ten neurons per animal were analyzed. Scale Bar = 50 µm. Error bar = SEM. (A–E) P-value = *<0.05, **<0.01, ***<0.001, (G) P-value = *<0.05 compared to control, +<0.05 compared to SOD1^G93A^.

**Figure 2:**
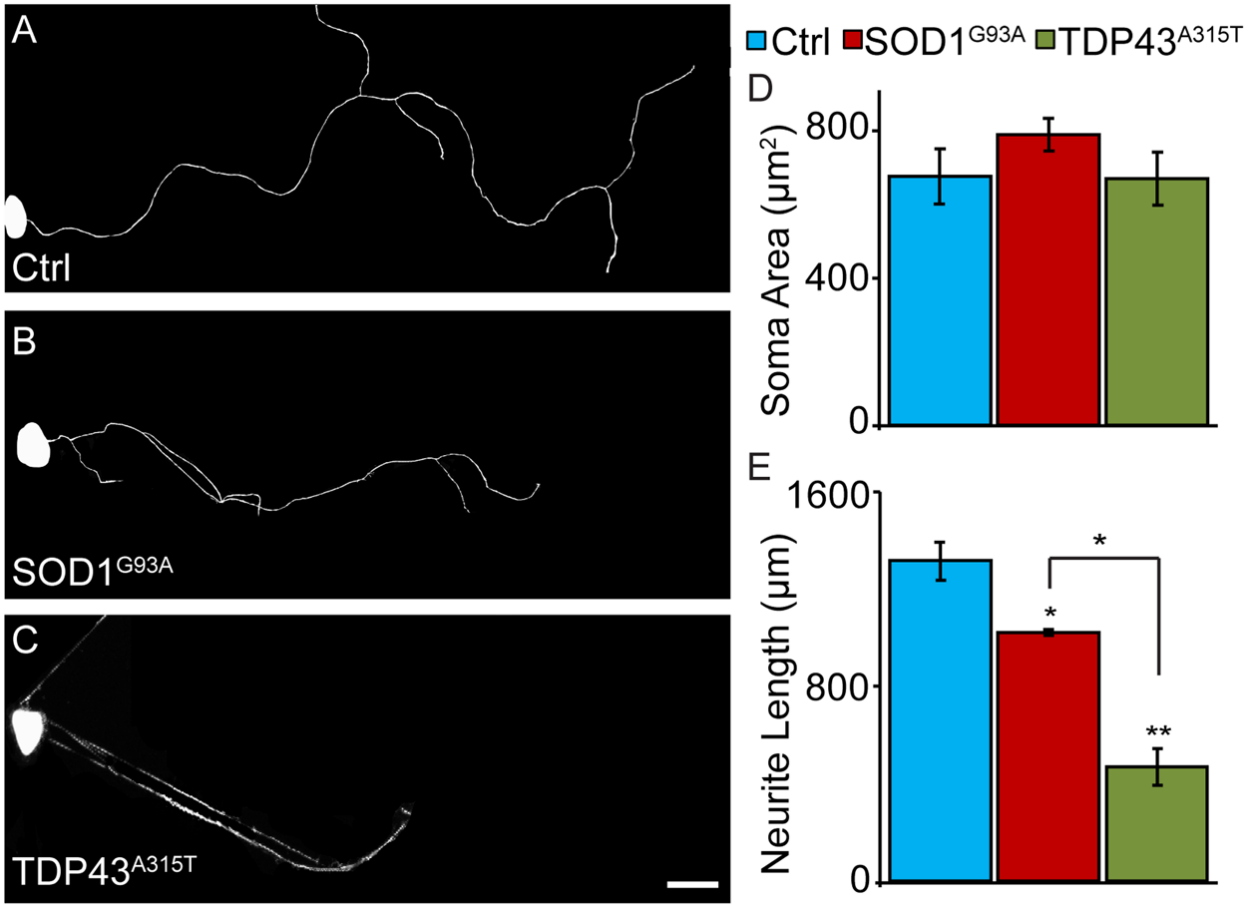
Decreased neurite length in SOD1^G39A^ and TDP43^A315T^ sensory neurons after 6 days in culture. Cultures of dissociated sensory neurons were examined after 6 days *in vitro*. Control (**A**) and SOD1^G93A^ (**B**) mice were crossed with Thy1-YFP16 transgenic animals expressing YFP to visualize sensory neurons. Sensory neurons from TDP43^A315T^ animals were visualized using anti-β-tubulin (**C**). The soma area of sensory neurons remains consistent across genotypes (**D**). However, sensory neurons from SOD1^G93A^ and TDP43^A315T^ animals show significantly decreased neurite lengths (**E**) compared to control. Control n = 3; 130 days old, SOD1^G93A^ n = 3; 130 days old, and TDP43^A315T^ n = 4; 130 days old. Only male mice were used for this experiment. At least five neurons per animal were analyzed. Scale Bar = 100 µm. Error bar = SEM. P-value = *<0.05, **<0.01.

We tested the possibility that TDP43^A315T^, a common mutation in familial ALS resulting in aberrant accumulation of TDP43 protein aggregates^25^, directly affects sensory neurons as demonstrated for SOD1^G93A^ using cultured sensory neurons. For this, we examined sensory neurons harboring TDP43^A315T^ in culture. We also analyzed sensory neurons expressing SOD1^G93A^ to compare the effects of these two ALS-causing mutations. Neurons were isolated from wild type mice and transgenic mice engineered to express either human TDP43^A315T^ or SOD1^G93A^, two well-characterized mouse models for ALS^26,27^. We first ascertained that TDP43^A315T^ and SOD1^G93A^ are expressed in dorsal root ganglia (DRG) and in cultured sensory neurons. As expected, both mutant genes were found to be expressed at high levels in both DRGs and in dissociated cultured sensory neurons. (see Supplementary Figs S1 and S2). Next, we analyzed the soma and neurites of cultured sensory neurons. We found that neither TDP43^A315T^ nor SOD1^G93A^ affect the soma size of sensory neurons cultured at low density for 24 hours (Fig. 1A–D). However, sensory neurons expressing either TDP43^A315T^ or SOD1^G93A^ have shorter neurites compared to control sensory neurons 24 hours post-culture (Fig. 1E). The presence of either mutant gene also reduced the complexity of their neuritic arbors, revealed using Sholl analysis (Fig. 1F–G). We then examined sensory neurons cultured for 6 days to assess their ability to grow long neurites and maintain them after an extended time in culture. We again found that sensory neurons expressing either SOD1^G93A^ or TDP43^A315T^ develop shorter neurites compared to control neurons (Fig. 2A–E) without a concomitant change in the soma size. Interestingly, these experiments show that sensory neurons with TDP43^A315T^ elaborate shorter and less complex neurites compared to sensory neurons expressing SOD1^G93A^ (Figs 1 and 2). What explains the more severe effect of TDP43^A315T^ compared to SOD1^G93A^ on sensory neurons? Among the many possibilities, the most parsimonious explanation is that TDP43^A315T^ has a more deleterious effect on molecular mechanisms, and thus subcellular structures, critical for the regeneration, maintenance, and repair of cultured sensory neurons.

### DRG explants from TDP43^A315T^ and SOD1^G93A^ mice fail to sustain the rate of neurite growth

We asked if TDP43^A315T^ and SOD1^G93A^ impair the ability of sensory neurons within whole DRGs to regenerate neurites. For this experiment, we repetitively imaged neurites extending from DRG explants isolated from lumbar region 3 (L3) of TDP43^A315T^, SOD1^G93A^ and wild type mice using bright-field imaging. DRGs were imaged at 72, 96, and 144 hours post-culture. Neurites were then traced from the middle of DRGs to the nerve ending to determine their length (See Supplementary Fig. S2). During the first 96 hours in culture, the rate of neurite outgrowth was similar between sensory neurons from all three genotypes (See Supplementary Fig. S2). By 144 hours in culture, the growth of neurites from sensory neurons harboring either TDP43^A315T^ or SOD1^G93A^ significantly slowed compared to control sensory neurons (See Supplementary Fig. S2).

**Figure 3:**
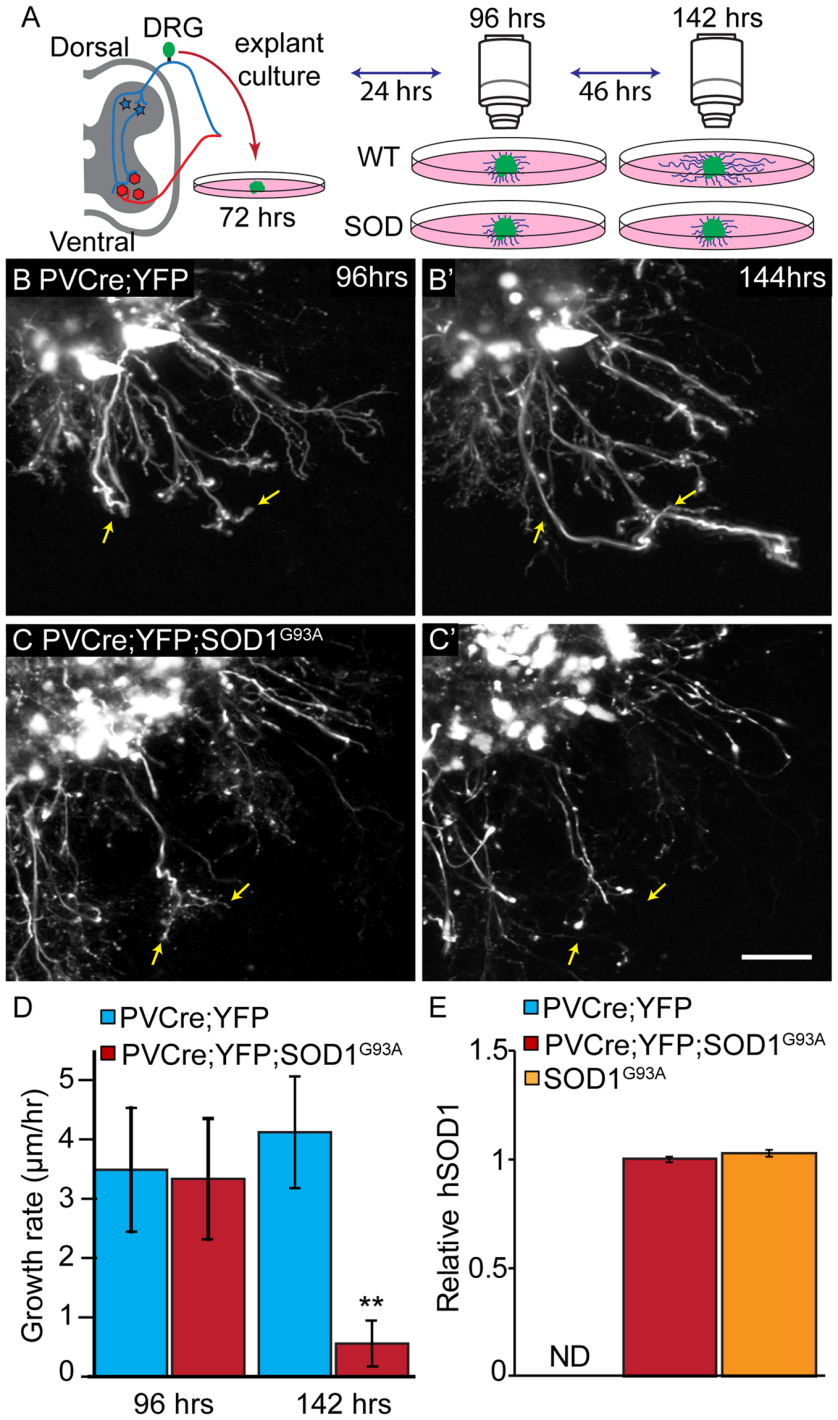
Sensory neurons from PVCre;STP-YFP;SOD1^G93A^ mice show decreased axonal growth rate. Explant cultures of PVCre;YFP and PVCre;YFP;SOD1^G93A^ DRGs were live-imaged at 72, 96 and 144 hours post-plating, and rates were determined based on the initial length at 72 hours (**A**). In control sensory neurons, the axonal growth rate remains consistent between 96 hours (**B**) and 144 hours (**B’**), while PVCre;STP-YFP;SOD1^G93A^ cultures show a significant decrease in growth rate between 96 hours (**C**) and 144 hours (**C’**,**D**). Arrows are marked in the same position at 96 hours (**B**,**C**) and 144 hours (**B’**, **C’**). hSOD1 mRNA is found at similar levels in DRGs from PVCre;YFP;SOD1^G93A^ compared to DRGs from SOD1^G93A^ mice and as expected not detected (ND) in wild type mice (**E**). Control n = 3; 130 days old, PVCre;STP-YFP;SOD1^G93A^ n = 3; 130 days old. At least four individual neurites were traced per explant with at least two explants examined per mouse. Represented values are the average of all animals per genotype. Only male mice were used for this experiment. Expression is normalized to GAPDH and relative to PVCre;YFP;SOD1^G93A^. Scale Bar = 100 µm. Error bar = SEM. P-value = **<0.01, ***<0.001.

To validate the findings described above, we live-imaged neurites extending specifically from proprioceptive sensory neurons within DRGs (Fig. 3A–C). We specifically examined proprioceptive sensory neurons because their afferents in muscle spindles have been shown to degenerate early and progressively in SOD1^G93A^ mice^23^. To image proprioceptive nerve endings, we generated mice expressing the yellow fluorescence protein (YFP) primarily in proprioceptive sensory neurons within DRGs. These mice were generated following the crossing of transgenic mice expressing Cre-recombinase from the parvalbumin locus (PV-Cre)^28^ with transgenic mice engineered to express YFP in neurons but only following the excision of a stop cassette flanked by LoxP sites^29^. Since the PV promoter is active in proprioceptive sensory neurons within DRGs^30^, the stop cassette is removed and YFP is expressed in these neurons. These mice (PV-Cre;STP-YFP) were then mated with SOD1^G93A^ mice to generate PV-Cre;STP-YFP;SOD1^G93A^ mice. As shown in Fig. 3E, SOD1^G93A^ is expressed at high levels in DRGs of PV-Cre;STP-YFP;SOD1^G93A^. To live image neurites, we isolated and cultured DRGs on 35 mm glass bottom dishes. During the first few days in culture, we observed no difference in the rate of axonal growth between control and SOD1^G93A^ expressing neurons (Fig. 3B–D). With additional time in culture, however, neurites extending from proprioceptive sensory neurons expressing SOD1^G93A^ grew significantly slower compared to controls (Fig. 3B’,C’,D). These data serve as additional evidence that ALS-causing mutant genes impair the ability of sensory neurons to regenerate neurites.

### TDP43^A315T^ and SOD1^G93A^ sensitize sensory neurons to the deleterious effects of vincristine

**Figure 4:**
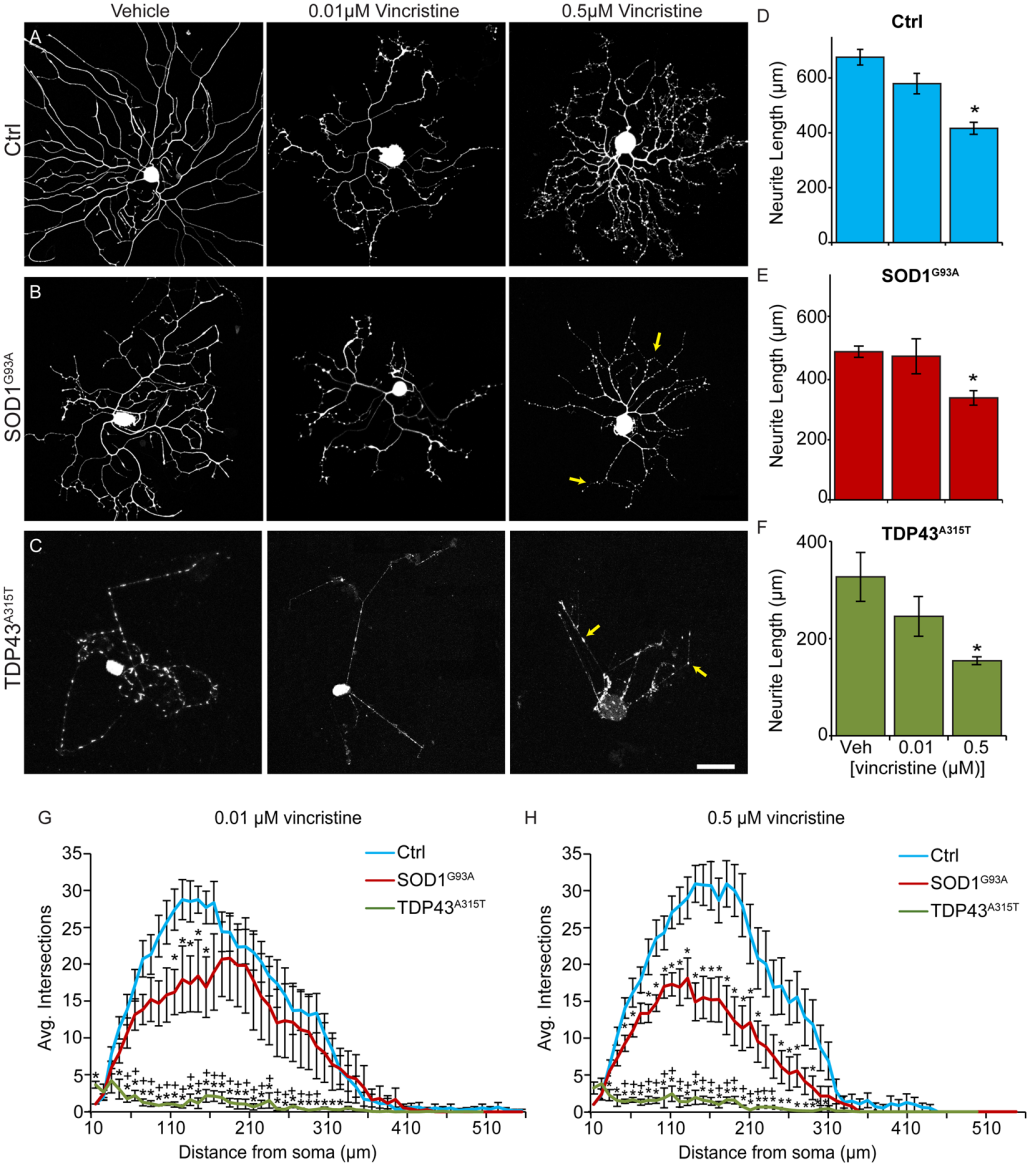
Sensory neurons from SOD1^G93A^ and TDP43^A315T^ mice have decreased neurite length and complexity following vincristine exposure. Dissociated cultures of DRGs from control (**A**), SOD1^G93A^ (**B**), and TDP43^A315T^ (**C**) mice were treated with 0.01 µM or 0.5 μM vincristine for 4 hours beginning 24 hours post-plating. Following exposure to 0.5 μM vincristine for 4 hours, control, SOD1^G93A^ and TDP43^A315T^ sensory neurons have significantly decreased neurite length compared to vehicle treated neurons (**D**–**F**). Sensory neurons from SOD1^G93A^ mice treated with 0.01 µM vincristine have significantly decreased neurite intersections between 110 and 130 µm from the soma compared to control (**G**). Sensory neurons from TDP43^A315T^ mice treated with 0.01 µM vincristine have significantly decreased neurite intersections between 30 and 330 µm from the soma compared to control (**G**). Exposure to 0.5 μM vincristine for 4 hours significantly decreases the number of neurite intersections between 50 and 290 µm in SOD1^G93A^ sensory neurons compared to control (**H**). Sensory neurons from TDP43^A315T^ mice treated with 0.01 µM vincristine have significantly decreased neurite intersections between 30 and 310 µm from the soma compared to control (**H**). Arrows (**B**,**C**) indicate blebbing neurites. Control n = 3; 130 days old, SOD1^G93A^ n = 3; 130 days old, and TDP43^A315T^ n = 4; 130 days old. Only male mice were used for this experiment. At least 5 sensory neurons were analyzed per animal. Represented values are an average of values from all animals per condition. Scale Bar = 100 µm. Error bar = SEM. (D–F) P-value = *<0.05, (G–H) P-value = *<0.05 compared to control, +<0.05 compared to SOD1^G93A^.

*In vivo*, mitochondrial dysfunction, accumulation of glial-derived toxic factors, and altered neurotransmission compromise the ability of neurons harboring ALS-inducing factors to maintain their axons^31^. The axons of ALS-affected neurons may also degenerate potentially due to instability of microtubules, a subcellular change that would also impair retrograde transport of pro-survival signals^31,32^. We therefore sought to determine if cultured sensory neurons expressing TDP43^A315T^ or SOD1^G93A^ are more susceptible to vincristine, a microtubule-destabilizing drug shown to cause progressive degeneration of neurites at low concentrations^33^. Sensory neurons were sparsely cultured for 24 hours and then exposed to vincristine at 0.01 µM and 0.5 µM. Neurons were then fixed and imaged at 4 hours post-treatment (Fig. 4A–C). We found that vincristine at 0.01 μM did not alter the length of sensory neurons’ neurites compared to vehicle with and without ALS-inducing factors (Fig. 4D–F). In contrast, vincristine at 0.5 μM significantly reduced the length of neurites of sensory neurons with and without ALS-inducing factors (Fig. 4D–F). We next compared the effect of vincristine on the neuritic arbors of sensory neurons with and without ALS-inducing factors using Sholl analysis. Both concentrations of vincristine reduced the number of neuritic branches on sensory neurons expressing either TDP43^A315T^ or SOD1^G93A^ compared to control neurons (Fig. 4G,H). Additionally, and supporting these findings, sensory neurons expressing either TDP43^A315T^ or SOD1^G93A^ qualitatively exhibited more blebs, which are varicosities that form along degenerating axons and dendrites^34^, compared to control in the presence of vincristine (Fig. 4A–C). These findings demonstrate that TDP43^A315T^ and SOD1^G93A^ increase the susceptibility of cultured sensory neurons to stressors. These experiments again revealed differences between sensory neurons harboring TDP43^A315T^ and SOD1^G93A^. Compared to SOD1^G93A^, the neurites of sensory neurons harboring TDP43^A315T^ are shorter and have fewer braches in the presence of vincristine (Fig. 4G,H).

### ATF3 and PERK levels correlate with TDP43^A315T^-induced pathogenesis *in vivo* and *in vitro*

**Figure 5:**
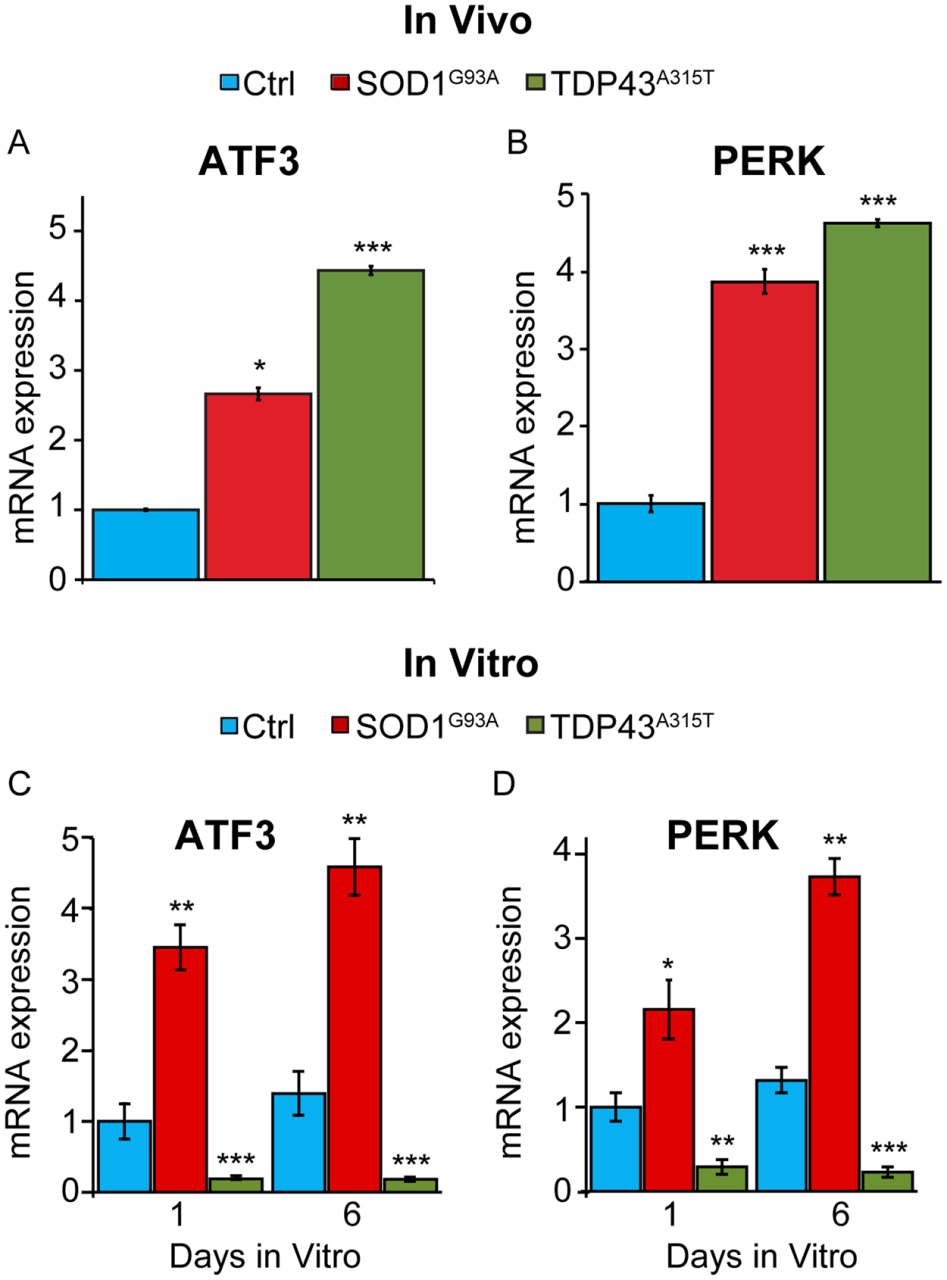
Differences in ATF3 and PERK expression in SOD1^G93A^ and TDP43^A315T^ mutant mice. ATF3 transcript levels from L2-L4 DRGs were quantified with qPCR and found to be significantly increased in SOD1^G93A^ and TDP43^A315T^ (**A**). A similar pattern of expression is seen in PERK from DRGs of SOD1^G93A^ and TDP43^A315T^ mice (**B**). In cultured sensory neurons, ATF3 and PERK mRNA expression is significantly increased in SOD1^G93A^ at both 1 and 6 days *in vitro* (**C**). However, ATF3 and PERK expression is significantly decreased in TDP43^A315T^ DRGs at both 1 and 6 days *in vitro* (**D**). Expression is normalized to GAPDH and relative to control condition, 1 day *in vitro*. *In vivo*: control n = 3; 120 days old, SOD1^G93A^ n = 3; 120 days old, and TDP43^A315T^ n = 5; 130 days old. *In vitro:* control n = 3; 120 days old, SOD1^G93A^ n = 3; 120 days old, and TDP43^A315T^ n = 5; 130 days old. Error bar = SEM. P-value = *<0.05, **<0.01, ***<0.001.

The activating transcription factor-3 (ATF3)^35,36^ and the protein kinase RNA-like endoplasmic reticulum kinase (PERK)^37,38^ are recruited to mitigate the deleterious effects of mutant SOD1 on neurons. ATF3 is increased in sensory and motor neurons to allow for the timely and proper regeneration of axons following injury^39–42^. It is also increased in sensory and motor neurons in SOD1^G93A^ mice^35^. Recently, ATF3 overexpression was shown to slow the degeneration of motor neurons in SOD1^G93A^ mice^36^. PERK is a critical component of the unfolded protein response (UPR) in the endoplasmic reticulum (ER), and implicated in ALS pathogenesis in mice expressing mutant SOD1^37,38^. We therefore asked if TDP43^A315T^ has a similar effect on levels of ATF3 and PERK as mutant SOD1 *in vivo* and *in vitro*. *In vivo*, we found that ATF3 and PERK mRNAs are significantly upregulated in L2-L4 DRGs from TDP43^A315T^ and SOD1^G93A^ mice compared to controls (Fig. 5A.B). We then visualized ATF3 in DRG sections from TDP43^A315T^, SOD1^G93A^ and control mice (See Supplementary Fig. S3). In DRGs from TDP43^A315T^ and SOD1^G93A^ mice, there are significantly more sensory neurons with ATF3 accumulated in the nucleus compared to control mice (See Supplementary Fig. S3). These findings show that sensory neurons harboring TDP43^A315T^ increase expression of two stress response molecules implicated in mitigating ALS-induced damages. They are also in agreement with published data showing increased levels of ATF3 in sensory neurons^43^ harboring SOD1^G93A^.

We next examined ATF3 and PERK transcripts in dissociated sensory neurons cultured for 1 and 6 days (Fig. 5C,D). As *in vivo*, sensory neurons harboring SOD1^G93A^ express higher levels of ATF3 and PERK compared to controls (Fig. 5C,D). In stark contrast, ATF3 and PERK are significantly reduced in sensory neurons harboring TDP43^A315T^ compared to control sensory neurons (Fig. 5C,D). These findings suggest that sensory neurons harboring TDP43^A315T^ lose the capacity to activate stress response mechanisms in culture, and largely in the absence of non-neuronal cells. They also partly explain why cultured sensory neurons harboring TDP43^A315T^ fail to regenerate neurites at a similar rate and are more sensitive to vincristine as those harboring SOD1^G93A^.

## Discussion

The data in this paper show that TDP43^A315T^ directly affects sensory neurons. In culture, sensory neurons expressing TDP43^A315T^ or SOD1^G93A^ grow and elaborate neuritic branches at a slower rate compared to control neurons. Additionally, we show that sensory neurons expressing TDP43^A315T^ or SOD1^G93A^ are more sensitive to vincristine, a microtubule-destabilizing drug that induces cellular stress. The presence of these ALS-causing mutant genes also affects expression of two stress response genes, ATF3 and PERK. These findings are significant since they indicate that intrinsic changes may be a driving force for the degeneration of sensory axons and their nerve endings in ALS.

Following dissociation, sensory neurons must regenerate their axons, called neurites in culture, a process that requires active and effective movement of cargoes to and from growing neurites^44^. In this regard, TDP43 and SOD1 have been shown to be involved in axonal transport. Wild type TDP43 is involved in axonal transport through its association with various cytoskeletal proteins. These include peripherin, a type III intermediate filament primarily expressed in neurons of the peripheral nervous system, the microtubule-associated protein 1B (MAP1B), neurofilament, and profilin^45^. Wild type TDP43 interacts with these cytoskeletal proteins to traffic granules containing RNA-binding proteins (RBPs) along axons^46^. However, mutant TDP43 cannot interact with these cytoskeletal proteins, inhibiting anterograde transport of RBP-positive granules and other cargoes along axons^45,47^. Highlighting the importance of TDP43 interaction with cytoskeletal proteins and axonal transport on maintaining axonal health and in ALS, mutated TDP43 was shown to cause the accumulation of RBP-positive granules in the cytosol of drosophila motor neurons and degeneration of axons^47^. Furthermore, mutant profilin has been shown to augment the deleterious effects of TDP43^A315T^ in addition to causing ALS-related pathogenesis on its own^48–51^. Thus, there is now ample evidence indicating that mutant TDP43 promotes ALS-pathogenesis by failing to interact with key cytoskeletal proteins and inhibiting transport of granules to axons. There is also evidence indicating that mutations in SOD1 cause ALS-pathogenesis partly by impairing retrograde transport. Specifically, SOD1^G93A^ has been found to interact with and inhibit the dynein-dynactin motor complex^52^. Additionally, retrograde transport has been shown to slow down in neurons harboring SOD1^G93A53^. While the experiments in this manuscript do not assess axonal transport and the integrity of the cytoskeleton, sensory neurons have been found to exhibit cytoskeletal disruptions in spinal muscular atrophy^54,55^, raising the possibility that a similar subcellular change may underlie their degeneration in ALS.

This study also revealed marked differences between sensory neurons expressing TDP43^A315T^ and SOD1^G93A^. Whether completely dissociated or within DRGs, sensory neurons expressing TDP43^A315T^ grow neurites at a slower rate compared to sensory neurons expressing SOD1^G93A^. The presence of TDP43^A315T^ also further sensitizes neurites to the adverse effects of vincristine. Additionally, cultured sensory neurons harboring TDP43^A315T^ express ATF3 and PERK at significantly lower levels compared to control neurons. In stark contrast, these two stress response genes are instead induced in cultured sensory neurons harboring SOD1^G93A^ compared to control neurons. Thus, the diminished expression of ATF3 and PERK may contribute to the accumulation of pathological features caused by TDP43^A315T^ expression in sensory neurons. Conversely, the effects of mutant TDP43 on neurites as well as on ATF3 and PERK expression may be due to loss of stress granules and deficits in RNA metabolism^46,56,57^, cellular and molecular actions required for neurons and other cells to adequately mount stress responses. However, these findings must be interpreted with caution since mutant TDP43 expression is driven from the prion promoter^26^ while multiple copies of mutant SOD1^27^ were introduced into cells. These two mutant genes are also expressed at very high levels in sensory neurons, potentially leading to adverse physiological effects unrelated to ALS-pathogenesis.

Our lab and others have shown that sensory nerve endings in the periphery degenerate early and progressively in SOD1^G93A^ and TDP43^A315T^ mice^26,27,58^. These cellular features are also found in motor neurons afflicted with ALS^59^. These findings suggest that similar molecular mechanisms with critical functions in maintaining and repairing axons and their nerve endings may become dysfunctional in both types of neurons. Thus, molecular analysis of these two neuronal subtypes should be undertaken in future experiments to uncover mechanisms that function to maintain and repair their respective nerve endings. Such molecular mechanisms could be therapeutic targets for preventing, slowing and even reversing ALS-pathogenesis.

## Materials and Methods

### Source of mice

We obtained SOD1^G93A^ mice^27^ (B6SJL-Tg(SOD1*G93A)1Gur/J) from Jackson Laboratories. Thy1-YFP16 (referred to here as Thy1-YFP) mice were a gift from Dr. Joshua R. Sanes. We generated mice expressing Thy1-YFP16 and SOD1^G93A^ and used littermates expressing Thy1-YFP16 as controls to visualize sensory neurons *in vitro*. These colonies are maintained in our animal facility on a mixed background. TDP43^A315T^ mice from Jackson Laboratories (B6.Cg-Tg(Prnp-TARDBP*A315T)95Balo/J)^26^ were obtained from ALS Therapy Development Institute via Charles River. Hydrogel packets were placed in the cages of TDP43^A315T^ animals to ameliorate sudden death from intestinal obstructions^60^. Controls used for comparison with TDP43^A315T^ animals were age-matched C57BL/6 mice. To exclusively visualize proprioceptive sensory neurons, Parvalbumin-Cre (referred here as PVCre)^28^ and Thy1-STOP-YFP line 15 (referred here as STP-YFP)^29^ mice were obtained from Jackson Laboratories and mated to generate PVCre;STP-YFP mouse expressing YFP selectively in PV neurons^23^. We then mated PVCre;STP-YFP animals with SOD1^G93A^ mice (referred to as PVCre;STP-YFP;SOD1^G93A^). To validate that the hSOD1 copy number remained similar in this new animal model, we performed copy number analysis from DNA of PVCre;STP-YFP;SOD1^G93A^ relative to SOD1^G93A^ DNA. In brief, DNA isolation was performed with the DNeasy Blood and tissue kit (Qiagen). Changes in transgene copy numbers were evaluated using quantitative real-time PCR by determining the difference in threshold cycle (ΔCT) between hSOD1 and the internal positive control gene. The probe for the transgene (hSOD1) was 13854, 5′-CTGCATCTGGTTCTTGCAAAACACCA-3′. The probe for the internal positive control was TmoIMR0105, 5′-CCAATGGTCGGGCACTGCTCAA-3′. Both sets of probes are described in the Jackson Laboratories protocol for genotyping hSOD1 transgenic mice. For each animal, 5 ng of genomic DNA was analyzed by the Bio-Rad CFX Connect Real-Time System (Bio-Rad) using TaqMan Universal PCR Mastermix (Thermo Fisher Scientific). The PCR was performed under the following conditions: 50 °C for 2 min, 95 °C for 10 min, 40 PCR cycles of 95 °C for 15 s, 60 °C for 1 min. The 2^ΔΔCT^ method was used to compare relative amounts between SOD1^G93A^ and PVCre;STP-YFP;SOD1^G93A^, which resulted in no significant differences. Only male mice were used for this study. Control data between wild type mice on a mixed background, Thy1-YFP and C57BL/6 mice are represented as one value in figures as they were not significantly different. Mice were anesthetized using isoflurane and either dissected immediately for cell cultures and fresh tissue or perfused transcardially with 1xPBS (pH 7.4) and 4% paraformaldehyde (PFA) for fixed tissue. All experiments were carried out under NIH guidelines and animal protocols were approved by the Virginia Tech Institutional Animal Care and Use Committee.

### Immunohistochemistry of DRG sections

Spinal columns were dissected immediately after perfusion and post-fixed in 4% PFA overnight at 4 °C. Following post-fixation, the spinal column was washed three times with 1xPBS and then cut below the last rib to separate the lumbar and sacral regions from the thoracic and cervical regions. The DRGs at L2 were dissected and incubated in 30% sucrose overnight at 4 °C. DRGs were then placed in Fisherbrand base molds with Tissue Freezing Medium (Triangle Biomedical Sciences, Inc.). Using a cryostat, 14 µm sections were collected on gelatin-coated slides. After washing with 1xPBS, sections were blocked for one hour with 0.1% Triton X-100, 4% BSA, and 5% lamb serum diluted in 1xPBS. Slides were then incubated with primary antibodies overnight at 4 °C. Following incubation, the slides were washed three times with 1xPBS for five minutes each. Sections were then incubated with secondary antibodies at room temperature for two hours. Slides were then washed three additional times with 1xPBS, incubated with DAPI (1:1000) for ten minutes and mounted using Vectashield (Vector Labs). The following primary antibodies diluted in blocking solution were used to label DRG sections: NeuN (Millipore MAB377; 1:250), Activating Transcription Factor-3 (ATF3, Santa Cruz Biotechnology sc-188; 1:50); the following secondary antibodies from Life Technologies were diluted 1:1000 in blocking solution: Alexa-488 goat anti-mouse IgG1, Alexa- 568 goat anti-rabbit. From the NeuN positive sensory neurons in the DRG, the percentage of neurons with ATF3 colocalized to DAPI was calculated as a percentage of total neurons with at least 30 neurons analyzed per animal. The represented values are the mean of four animals per genotype.

### Culture of dissociated sensory neurons

DRGs were dissected from control, SOD1^G93A^, and TDP43^A315T^ then kept in HBSS (Life Technologies 14175103) on ice. After the connective tissue and axonal stumps were removed, DRGs were digested in papain (40 U/ml; Worthington 9001- 73-4) for 10 min, collagenase II (10 mg/ml; Worthington LS004177) and dispase (5 U/ml; StemCell Technologies Inc. 07913) for 15 min at 37 °C, and triturated with a glass Pasteur pipette ten times in Neural Basal A (NBA; LifeTechnologies 10888022) media supplemented with 10% FBS. Dissociated cells were then passed through a 70 µm cell strainer, plated at 25 K/mL on Flexiperm (Sarstedt Inc. 94.6032.039) chambers on Permanox (Nunc 160005) slides coated with Matrigel (1:10 in NBA media; Fisher Scientific CB40234) and cultured in NBA media supplemented with B27 (LifeTechnologies 17504044).

### Immunohistochemistry of sensory neuron cultures

Half of the media was removed from the culture dish and replaced with an equal volume of 8% PFA and 30% sucrose solution. After a 30-minute incubation, the chambers were washed three times with 1xPBS for 5 minutes each. The chambers were blocked for one hour with 0.05% Triton X-100, 4% BSA, and 5% lamb serum diluted in 1xPBS. Chambers were then incubated with primary antibodies overnight at 4 °C. Following incubation, the chambers were washed three times with 1xPBS for five minutes each. Chambers were then incubated with secondary antibodies at room temperature for two hours. Slides were washed three additional times with 1xPBS, incubated with DAPI (1:1000) for ten minutes and mounted using Vectashield (Vector Labs). The following primary antibodies diluted in blocking solution were used to label sensory neurons in culture: Beta tubulin-III (Sigma Aldrich T2200; 1:1000). The following secondary antibodies from Life Technologies were diluted 1:1000 in blocking solution: Alexa-488 goat anti-rabbit.

### Neurite and soma size analysis

Neurites that appeared to be well established were traced and measured using NeuronJ of ImageJ. The longest neurite extending from each neuron was used to calculate the average *longest neurite length* for each animal. The area of each soma was traced and measured in ImageJ. At least 15 neurons were analyzed per animal. The average values for each treatment category is represented as the average of the values from all animals in the respective treatment category.

### DRG explant culture

DRGs were dissected and kept in HBSS (Life Technologies 14175103) on ice until digestion with collagenase II (10 mg/ml; Worthington LS004177) and dispase (5 U/ml; StemCell Technologies Inc. 07913) for 5 min at 37 °C. After washing three times with NBA media supplemented with B27, DRGs were plated in Flexiperm chambers on Permanox slides initially coated Matrigel (1:10 in NBA; Fisher Scientific CB40234) and then covered with a drop of undiluted Matrigel to help DRGs attach to the dish. DRGs were maintained in NBA media supplemented with B27 throughout all imaging sections.

### Imaging neurite growth rate

DRGs that appeared well-attached to the dish with large neuritic arbors were imaged and others were discarded. Seventy-two hours post-plating, DRG explants were imaged at indicated time points (72, 96, and 144 hrs). DRGs from 50 day-old C57BL/6, TDP43^A315T^, and SOD1^G93A^ were imaged using a Leica DMLB transmitted light microscope with a 40X water-immersion objective using LASV4.3 software. Multiple images were taken of each DRG in different areas at each time point. Neurite length was measured using NeuronJ, a plugin for ImageJ. At least 20 neurites were analyzed per DRG at each time point and at least five DRGs per animal The average neurite length of all neurons from each animal was calculated. The represented values are an average of 3 animals of the same genotype in each treatment category. DRGs from PVCre;STP-YFP and PVCre;STP-YFP;SOD1^G93A^ animals were live imaged using a Zeiss LSM 700 motorized confocal microscope equipped with a Hamamatsu Flash 4.0 camera. One static image was taken for each time point, and images from all time points were merged sequentially in ImageJ. To trace neurite growth, drift was removed from image sequences, and neurites that could be distinguished and tracked throughout all time points were traced with the NeuronJ plugin, their lengths measured, and rates determined by dividing the difference in length by time interval between imaging. At least 4 neurites were traced per DRG explant and 2 explants per animal. The average growth rate was calculated for each animal. The represented values are an average of 3 animals of the same genotype.

### Vincristine treatments

Cultures were treated by replacing half the media volume with either 0.02 µM or 1 µM vincristine (Sigma Aldrich v8879) diluted in culture media. In control conditions, half of the media volume was replaced with fresh media with <0.1% DMSO. After four hours of vincristine exposure, chambers were fixed, following the protocol described above, and analyzed.

### Sholl analysis

Using images with a single neuron in the frame, we conducted Sholl Analysis using ImageJ after adjusting the image threshold accordingly. The segmented line tool was used to determine both the center of the soma and the length of the largest integration radius. The parameters were set to allow for continuous measurements, and the data were fit to a linear polynomial function of the best fitting degree. Data were grouped with their respective categories (vehicle, 0.01 µM and 0.5 µM). At least 5 neurons were analyzed per animal with at least 3 animals examined per genotype. The average number of neurite intersections from control, SOD1^G93A^ and TDP43^A315T^ neurons at each radius was plotted according to the treatment they received.

### Expression analysis using quantitative PCR (qPCR)

Mice were first anesthetized with isoflurane then DRGs from lumbar and sacral regions and TA muscles were dissected. For expression analysis, DRGs and TA muscles were immediately flash frozen in liquid nitrogen. Total RNA was prepared using Aurum Total RNA Mini kit (Bio-rad) following the manufacturer’s instructions. Then cDNA was synthesized from 100 ng of total RNA using the iScript cDNA synthesis kit (Bio-rad). PCR amplification was performed on the Bio-Rad CFX Connect Real-Time System (Bio-Rad) using iTaq Universal SYBR Green Supermix (Bio-rad). All primers used in this study are listed in Table 1.

**Table 1:** qPCR Primers.

Gene	Fw (5′-3′)	Rv (5′-3′)
GAPDH	CCCACTCTTCCACCTTCGATG	GTCCACCACCCTGTTGCTGTAG
ATF3	GAGCTGAGATTCGCCATCCA	CCGCCTCCTTTTGCCATCCA
PERK	TCTTGGTTGGGTCTGATGAAT	GATGTTCTTGCTAGTGGGGG
hSOD1	GTGTGGCCGATGTGTCTATT	TTTCATGGACCACCAGTGTG
hTDP43	CCATCGGAAGACGATGGGAC	TGGGGCATGCAGAATTCCTT

### Statistics

For experiments with three experimental groups, a one-way ANOVA with Bonferroni post-hoc was used to determine statistical significance between treatment groups. For comparisons between two experimental groups, a student’s t-test was used to determine significance. For Sholl analyses, an ANOVA was used to determine significance between the three genotypes, followed by a student’s t-test assuming unequal variance to determine significance between the number of intersections from control and SOD1^G93A^, control and TDP43^A315T^, and SOD1^G93A^ and TDP43^A315T^ at each intersection radius. Bar graphs are represented as mean ± standard error. Statistical analysis was performed using R statistics and a p value < 0.05 was considered significant.

## Electronic supplementary material

Supplementary Figures
