# Supplementary material for: The ALS-inducing factors, TDP43A315T and SOD1G93A, directly affect and sensitize sensory neurons to stress
Source: Sci Rep. 2018 Nov 8;8:16582. doi: 10.1038/s41598-018-34510-8 (PMC6224462; doi:10.1038/s41598-018-34510-8)
Supplement: Supplementary file 1 — Supplementary Figures [file 41598_2018_34510_MOESM1_ESM.pdf]

The ALS-inducing factors, TDP43^{A315T} and SOD1^{G93A}, directly affect and sensitize sensory neurons to stress

Sydney K. Vaughan[1,2], Natalia M. Sutherland[1], Sihui Zhang[1], Theo Hatzipetros[4], Fernando Vieira[4], Gregorio Valdez[1,3]

[1]Virginia Tech Carilion Research Institute, Roanoke, Virginia, USA
[2]Graduate Program in Translational Biology, Medicine, and Health, Virginia Tech, Blacksburg, Virginia, USA
[3]Department of Biological Sciences, Virginia Tech, Blacksburg, Virginia, USA
[4]ALS Therapy Development Institute, Cambridge, MA USA

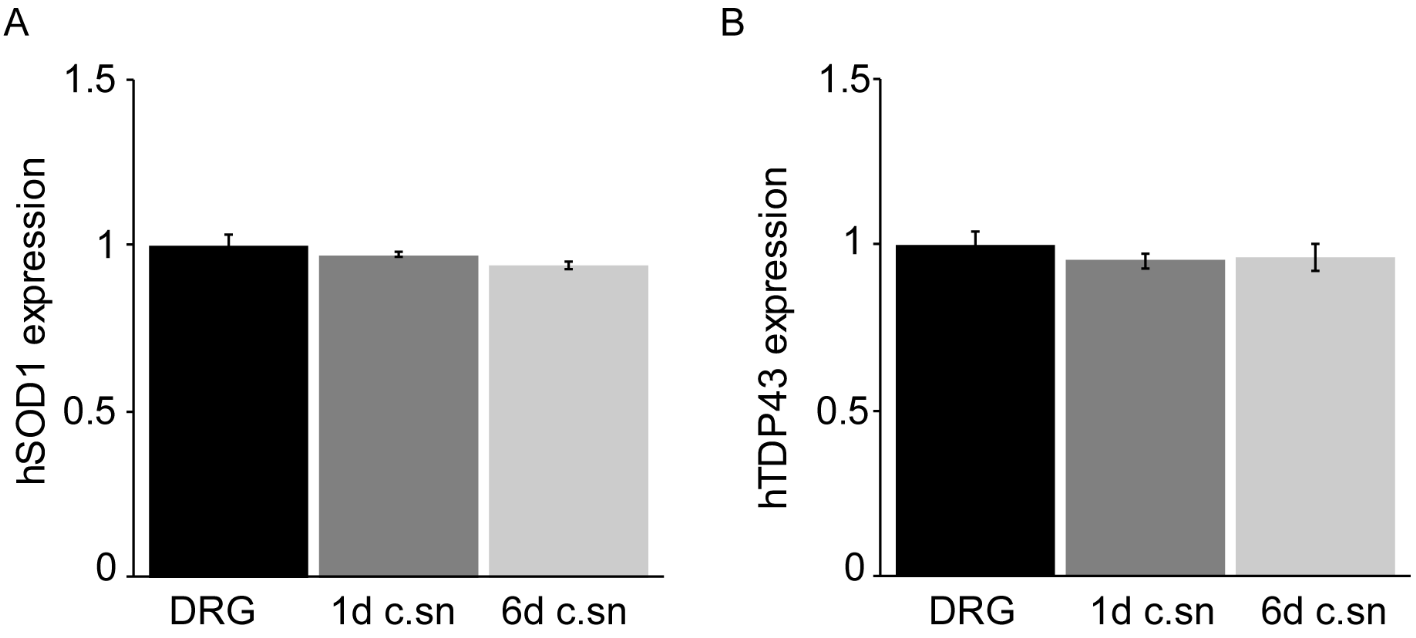

Fig. S1. hSOD1 and hTDP43 expression is upregulated in dissociated DRG cultures. Dissociated cultures of DRGs from control, SOD1^{G93A}, and TDP43^{A315T} mice were cultured for 1 and 6 days. At each time point, RNA was isolated from cultured neurons. hSOD1 mRNA expression is similar between whole DRGs and dissociated cultured sensory neurons (c.sn) dissociated from 120 days old SOD1^{G93A} mice and kept in culture for 1 and 6 days (A). hTDP43 mRNA expression is similar between whole DRGs and cultured sensory neurons (c.sn) from 130 days old TDP43^{A315T} mice and kept in culture for 1 and 6 days (B). Expression is normalized to GAPDH and relative to whole DRG. hSOD1 and hTDP43 were not detected in control animals. At least 3 DRGs and 5 biological replicates for cultured sensory neurons were examined for every comparisson. Error bar = SEM.

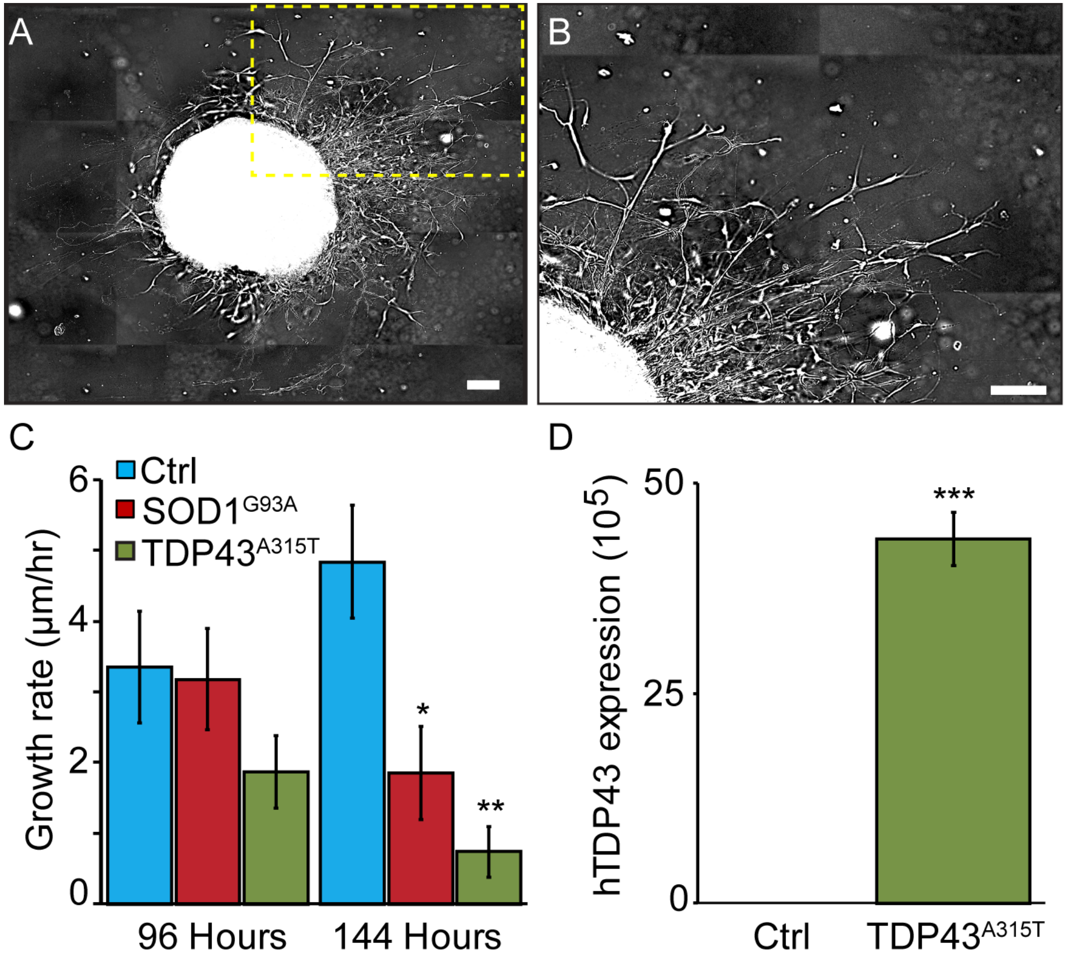

Fig. S2. Sensory neurons from TDP43^{A315T} and SOD1^{G93A} mice show decreased axonal growth rate. Explant cultures of control, TDP43^{A315T}, and SOD1^{G93A} DRGs from L3 were imaged in bright-field with 40x magnification at 72, 96 and 144 hours post-plating. The growth rate of individual neurites was determined based on their initial length at 72h. A representative control explant at 144 hours post plating (A) shows neurites extending from the DRG denoted by the dotted line, and enlarged in (B). All of the sensory neurons maintained a similar growth rate between 72 hours and 96 hours (C), while TDP43^{A315T} and SOD1^{G93A} cultures show a significant decrease in growth rate between 96 hours and 144 hours (C). The mRNA expression of hTDP43 is significantly upregulated in DRGs of TDP43^{A315T} compared to control DRGs at 130 days of age (D). At least 3 DRGs per genotype were examined and 20 neurites were traced per explant. Only 50 days old male mice were used for this experiment. Scale Bars (C) = 200μm. Error bar = SEM. P-value = * <0.05, **<0.01.

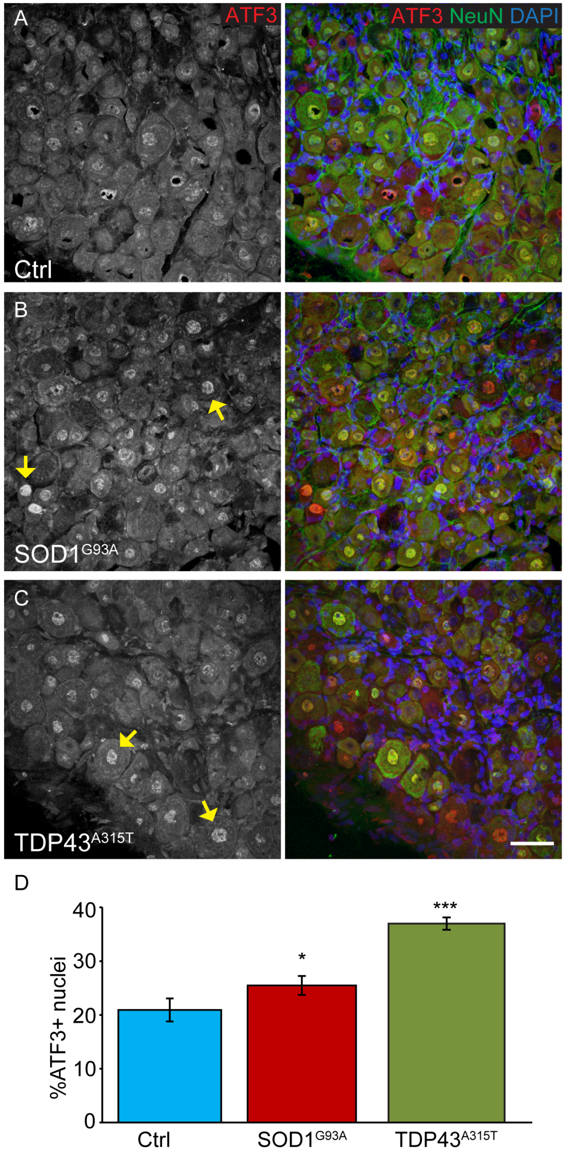

Fig. S3. Increase in ATF3⁺ Nuclei in DRGs from ALS-afflicted mice. L2 DRG slices from control (A), SOD1^{G93A} (B), and TDP43^{A315T} (C) were stained with ATF3 (red), NeuN (green), and DAPI (blue). Data show that the number of neurons with ATF3 localized to the nucleus is elevated in sensory neurons expressing mutant SOD1^{G93A} and TDP43^{A315T} when compared to control (D). Yellow arrows (B, C) show ATF3 localized to the nuclei of sensory neurons. Control n=4; 70 days old, SOD1^{G93A} n=4; 70 days old, TDP43^{A315T} n=4; 80 days old. Only male mice betwee 70-80 days of age were used for this experiment. At least 4 DRGs and 20 sections per DRGs and130 neurons per DRG were analyzed for each genotype. Scale Bar = 100µm. Error Bar = SEM. P-value =*<0.05, ***<0.001.
